# Measurement of micronutrient deficiency associated biomarkers in dried blood spots using a multiplexed immunoarray

**DOI:** 10.1371/journal.pone.0210212

**Published:** 2019-01-08

**Authors:** Eleanor Brindle, Lorraine Lillis, Rebecca Barney, Pooja Bansil, Christopher Lyman, David S. Boyle

**Affiliations:** 1 Center for Studies in Demography and Ecology, University of Washington, Seattle, Washington, United States of America; 2 PATH, Seattle, Washington, United States of America; 3 Quansys Biosciences, Logan, Utah, United States of America; University of North Carolina at Chapel Hill, UNITED STATES

## Abstract

Simplifying blood collection is often critical when collecting specimens in remote and/or austere settings. The use of dried blood spots (DBS) offers a practical collection method suitable for a wide variety of analytes. A small volume of whole blood can be obtained rapidly through a minimally invasive heel- or finger-stick using a disposable safety lancet. Once dried, the samples require no further processing, are stable for months or longer, pose minimal risk of disease transmission, and are easy to ship. DBS are often used in demographic health surveys to assess infectious disease status in vulnerable populations. These samples can be used to screen biomarkers of micronutrient deficiency (MND) and inflammation. We recently described a multiplexed immunoarray, the Q-plex human micronutrient array, which can simultaneously quantify seven biomarkers related to MND, inflammation and malarial antigenemia using plasma (alpha-1-acid glycoprotein, C-reactive protein, ferritin, histidine-rich protein 2, retinol binding protein, soluble transferrin receptor, and thyroglobulin). In this work, we present a protocol for preparing eluates from DBS samples and their measurement using a modified protocol for this new tool. We evaluated the concordance of analyte concentrations (excluding ferritin) from a panel ninety samples of DBS prepared from anticoagulated venous blood and paired K_2_-EDTA plasma. The results show high correlation between DBS eluates and wet plasma for five of the six analytes screened, suggesting the Q-plex human micronutrient array can be used with DBS samples, but also highlighting that anticoagulants can have a negative effects on some test components.

## Introduction

Micronutrient deficiencies (MND) have a significant impact on health by affecting cognitive, immune, ocular and physical health and development. Therefore, young children and women of reproductive age in low- and middle-income countries are particularly at risk. Iron, vitamin A and iodine are three of the primary MNDs of global significance. Iron affects the feto-maternal wellness in addition to severely restricting cognitive and physical development in infants and young children [1,2]. Vitamin A deficiency (VAD) negatively affects ocular development and can lead to blindness [3]. VAD is also thought to limit cellular and humoral immune responses contributing to greater risk of morbidity and mortality from infectious diseases in infants and young children [4]. Iodine deficiency, even mild, is believed to negatively affect cognitive development even in mild deficiency, and significant deficiency can result in goiter and other physical health problems [5].

There are low cost and effective solutions to address MND including dietary choices, fortification of foodstuffs or more directly via supplementation [6,7]. However, for these interventions to have optimal impact, population surveillance must be carried out prior to intervention to assess micronutrient status in order to identify at-risk populations and establish a baseline, and sequentially thereafter to determine intervention effectiveness. A series of blood-based biomarkers have been identified that can be used to inform on micronutrient status [8–12]. Use of these biomarkers simplifies population surveillance in austere environments as the quantitative assays used to assess these biomarkers involve simpler operating procedures and remove the need for highly complex equipment (e.g. high pressure liquid chromatography for vitamin A analysis) [13].

The blood specimens are collected either via venipuncture or less invasive methods, such as finger- or heel-stick blood collection using a lancet. Venous blood collection offers much larger sample volumes but these are more challenging to collect, process, and store. Whole blood must be rapidly processed to avoid hemolysis and the serum or plasma must then be kept in cold chain to limit or prevent decay of the biomarkers, making storage and shipping logistically challenging in austere or low resource environments. Participants may also have a preference for fingerstick over blood venous collection due to a perception of greater pain associated with the latter method [14].

Blood and its liquid derivatives also present a biohazard via blood-borne pathogens that remain infectious under cold chain storage. Dried blood spots (DBS), by comparison, can be collected by a less skilled user, present significantly reduced biohazard risk during handling and processing, and sample preparation takes less time and fewer materials [15–17]. The volume of capillary blood collected from a fingerstick is however much smaller (up to 250 μL [18]) and is then dried and stored in a sealed pouch with a desiccant to eliminate moisture. DBS cards have the further advantages of being relatively small and many analytes, including biomarkers for micronutrient status and inflammation, remain stable within DBS at higher temperatures than wet plasma or serum, therefore simplifying shipping and storage [12,19–22].

A challenge to using DBS as a sample type is that the amount of sample material is limited, with a typical collection yielding five spots of approximately 50 μL whole blood each. Additionally, elution of blood from the filter paper requires submerging measured quantities of blood-impregnated paper in an assay buffer, which dilutes the biomarkers. We recently described the development of the Quansys Biosciences Q-Plex Human Micronutrient Array (Logan, Utah, USA), a multiplexed immunoarray that enables the simultaneous detection of biomarkers of deficiencies in iodine, iron, and vitamin A, in addition to biomarkers of inflammation and *Plasmodium falciparum*, from a single small volume of liquid serum or plasma [23]. We hypothesize that the same assay could be used to accurately detect and quantify the biomarkers using DBS as the specimen type because simultaneous measurement of all these analytes in a single test requires a relatively small sample volume. In this study, we highlight the development of a DBS elution protocol and present correlative datasets from paired DBS and plasma samples from 90 participants. We demonstrate the potential of using DBS for the detection of relevant biomarkers for micronutrient status via a relatively simple but effective multiplexed immunoassay format.

## Materials and methods

### Donor blood panel

The specimens were procured from a commercial vendor. The PATH research ethics committee deemed this non human subjects research. A panel of 90 K_2_-EDTA (ethylene diamine tetra acetic acid) anticoagulated whole blood samples (45 adult male, 45 adult female) was procured from BioIVT Inc. (Westbury, New York, USA). Upon receipt of the whole blood, Whatman 903 cards were spotted with five 50 μL volumes of blood dropped from a pipette. DBS cards were stored at room temperature overnight to dry the blood, then individually stored at -80°C in sealed plastic pouches contacting desiccant packets. The remaining whole blood was treated as previously described to prepare plasma fractions [24]. The plasma fractions were aliquoted into cryovials and stored at -80°C until use.

### Preparation of contrived malaria-positive dried blood spots

As the set of paired plasma and DBS specimens prepared from US donor blood were all histidine-rich protein 2 (HRP2) negative, HRP2 recovery from DBS was tested using contrived DBS samples created via two methods. First, whole blood was collected by venipuncture in K2-EDTA coated tubes and centrifuged to recover the red blood cell (RBC) layer. RBCs were then washed three times in normal saline. Purified standard materials for all seven analytes in the 7-Plex array were combined and diluted in phosphate buffered saline (PBS) with 0.5% bovine serum albumin (BSA) to create six different concentrations of each analyte and one zero dose containing PBS/BSA only [23]. Each concentration was then combined with an equal volume of washed RBCs and 75 μL of each concentration/RBC mixture was pipetted onto Whatman 903 cards from a wide-bore pipet tip and the spotted cards then processed as previously described.

Malaria-positive DBS were also prepared from contrived whole blood samples made from a laboratory-adapted strain of *P*. *falciparum*, 3D7 grown in culture, and assessed for parasitemia via Giemsa stain of thick RBC smears and microscopy on the day of harvest [23]. Samples were combined with normal blood to create samples with high, medium, low, and negative % levels of all stages of parasitemia. These specimens were spotted onto Whatman 903 cards to prepare DBS as described above.

### Human micronutrient (7-Plex) assay procedure

The Quansys Q-plex protocol instructs the user to prepare all sample, negative control and calibrator dilutions in sample diluent containing reconstituted competitor. Because the competitor may degrade in the extended incubation time required for DBS elution, the standard protocol was modified for use with DBS. These protocol alterations were used for both the DBS and wet plasma specimens included in these analyses.

Two 6 mm punches, each containing approximately 6.1 μL of serum [16], were taken from each natural or contrived DBS specimen and submerged in 122 μL of kit diluent without competitor mixture added (hereafter “incomplete” diluent), and eluted overnight at 4°C to produce eluates equivalent to a 1:10 serum or plasma dilution. The next day, eluted specimens were allowed to mix on a microtiter plate shaker at 500 revolutions per minute for one hour at room temperature before use. Matching plasma specimens and a commercial quality control specimen (Liquiche Immunology Control Level 3, Bio-Rad, Hercules, California, USA) were also diluted 1:10 in incomplete kit diluent. Due to the extra dilution of the samples (1:20 versus 1:10), the standard curves were prepared in incomplete kit diluent at 2 times the concentrations suggested in the kit instructions. First, the lyophilized calibrator was reconstituted with half the volume of incomplete sample diluent suggested in the kit insert, then a further series of seven threefold dilutions was prepared to create an eight -point standard curve. (Note different calibrator lots may require different diluent volumes for rehydration). Next, the lyophilized competitor mix provided with the kit was reconstituted using half the suggested sample diluent volume recommended in the product insert to produce a 2X strength competitor mix. A 60 μL volume of each eluted DBS or diluted plasma sample or control (1:10) was combined with an equal volume of 2X competitor mix to produce final dilutions of 1:20 for each sample and control in a 1X competitor solution. Similarly, each standard curve point was combined with an equal volume of 2X competitor mix to achieve the suggested concentrations for each standard point in 1X competitor mix. A volume of 50 μL per well of standards, controls and samples were added to the plates in duplicate wells. From this point, the protocol proceeded as described in the kit instruction booklet.

After addition of the standards and samples, each plate was incubated at room temperature for two hours with shaking using a flatbed shaker (Titertek Berthold, Huntsville, Alabama, USA) at 500 RPM. All reactions were aspirated and washed 3 times with 400 μL of 1x Tris buffered saline Tween20 using an automatic plate washer (ELx50 plate washer, BioTek Instruments Inc., Winooski, Vermont, USA). Next, 50 μL of detection mix was added to each well and the plate was then incubated with shaking for 1 hour and washed one more time as described above. Labeling was performed by adding 50 μL streptavidin horseradish peroxidase solution to each well and shaking for 20 minutes, then the plate samples were aspirated and washed twice. The chemiluminescent substrate parts A and B were mixed in equal volumes and 50 μL of the mixture was then added to each well.

Each plate was then imaged at 270 seconds of exposure time using a Quansys Q-View Imager LS (Quansys Biosciences). Q-View Software (Quansys Biosciences) was used to overlay a plate map onto the locations of analyte spots in each well and to measure the chemiluminescent signal from each spot in units of pixel intensity. The software applies the calibrator concentration values to the pixel intensities for each spot in the standard curve wells and fits 5 parameter logistic calibration curves for each analyte. The pixel intensities of the spots in each test well are then used to interpolate the concentration of each analyte relative to its calibrator curve. Once the plate image is overlaid with the analysis grid, all of the curve fitting and data reduction steps are automatically applied via the software. The upper and lower limits of quantification determined by Quansys for each kit lot were applied to exclude values beyond the concentration ranges that yield precise concentration estimates. Values less than the reliably quantifiable concentration range for each analyte were excluded from analyses and values with concentrations exceeding the acceptable concentration ranges were assayed again at a final dilution of 1 in 40 (result greater than concentration limits for n = 3 DBS and plasma specimen pairs, thyroglobulin (Tg) values only). All values were adjusted for dilution used.

### Statistical methods

Ferritin results were excluded from analyses; DBS ferritin includes a mixture of ferritin from serum and ferritin released when red blood cells lyse, and thus is not directly comparable to plasma ferritin [20]. It has been noted that ferritin levels are elevated three fold when whole blood is the sample type [20]. HRP2 results from the panel of 90 samples from US donors were excluded from analyses because all samples were negative for HRP2. DBS HRP2 was thus evaluated only by recovery from contrived HRP2-positive DBS samples. Concordance of α-acid glycoprotein (AGP), C reactive protein (CRP), retinol binding protein 4 (RBP4), soluble transferrin receptor (sTfR) and thyroglobulin (Tg) results for 90 pairs of plasma and DBS was evaluated by Spearman correlations and by scatter plots and linear regression. Agreement in absolute value was evaluated by calculating by recovery of plasma values from DBS (DBS concentration/plasma concentration, expressed as a percentage) and using Bland Altman plots of the average concentration plotted against the percentage difference in concentrations of each analyte in plasma/DBS sample pair.

## Results

For the five analytes tested in the 90 specimen pairs (900 tests total), 883 results were within the calibration range when processed at a 1:20 dilution, rather than the 1:10 dilution recommended in the kit instructions. All test data can be publicly accessed at Dataverse (https://dataverse.harvard.edu/dataverse/biomarkers_micronutrient_immunoarray). All results were valid for three analytes: AGP, RBP4, and sTfR. For CRP, ten DBS specimens and one plasma specimen had results less than the lower limit of quantification. For Tg, four results (one DBS and three plasma) were less than the lower limit of quantification, and three were greater than the upper limit of quantification. The high Tg concentration samples were re-tested at a 1:40 dilution; one DBS/plasma pair, which remained above the upper limit of Tg quantification at 1:40, was excluded from subsequent analyses. Samples with CRP or Tg results below the lower limit of quantification at a 1:20 dilution were not retested as the sample could not be concentrated further to increase the concentration of analyte, and were excluded from analyses. For the five analytes included in analyses, average intra-assay CVs for duplicate well results from the 90 DBS specimens and 90 plasma specimens ranged from 3.3% to 4.9% and from 1.8% to 4.3%, respectively.

**Fig 1:**
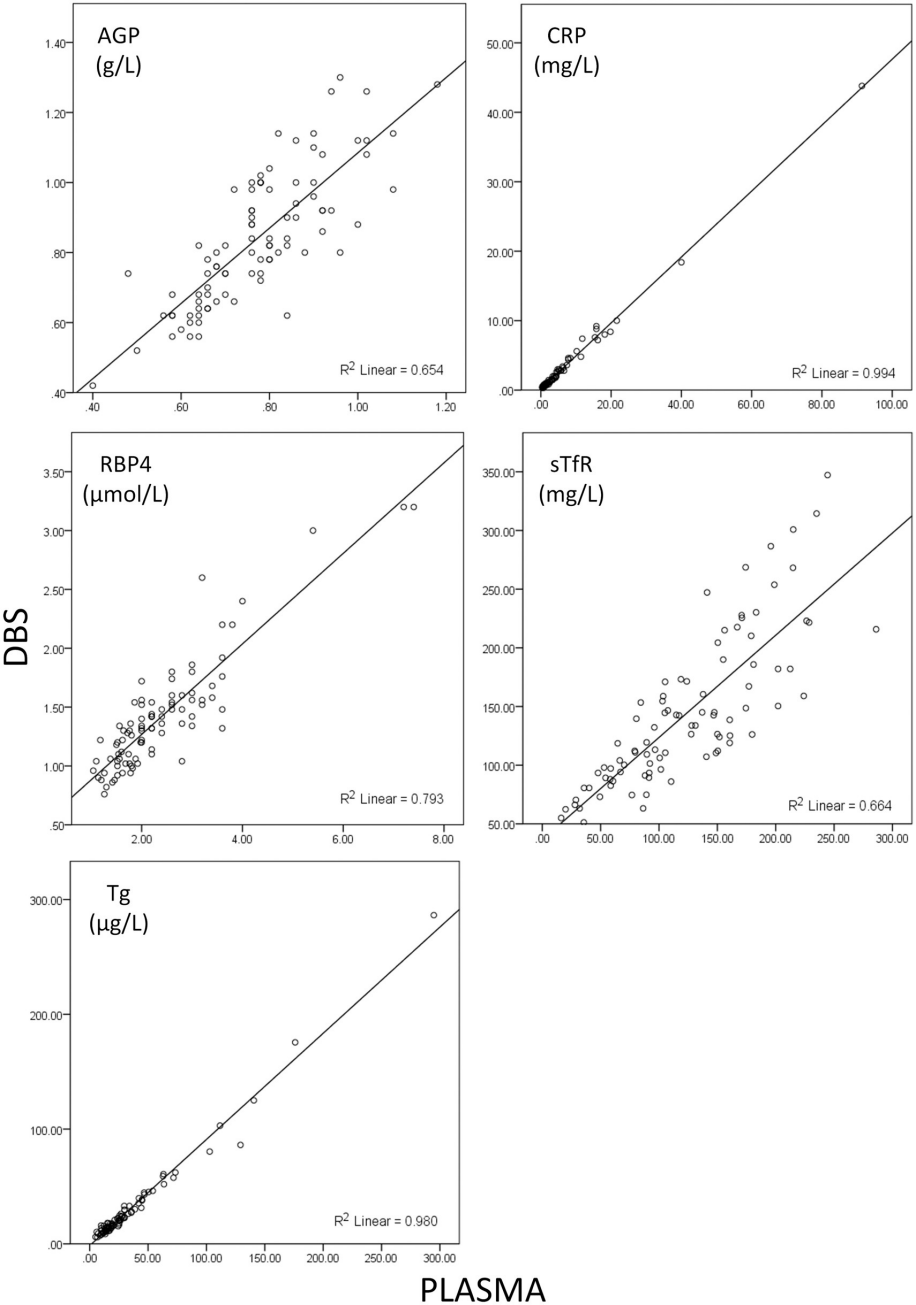
Scatter plots of the absolute values from DBS eluates versus paired wet plasma samples derived the human micronutrient Q-plex assay. Concentrations of each analyte derived from DBS as measured in the 7-Plex (y-axes) plotted against concentrations measured using paired wet plasma samples (x-axes). Solid line is linear least squares regression (y = mx+b). For CRP, 10 outliers were excluded from regression and for Tg 1 outlier was excluded from regression. AGP, α-1-acid glycoprotein; CRP, C-reactive protein; RBP4, retinol binding protein 4; sTfR, soluble transferrin receptor; Tg, thyroglobulin; DBS, dried blood spot.

**Fig 2:**
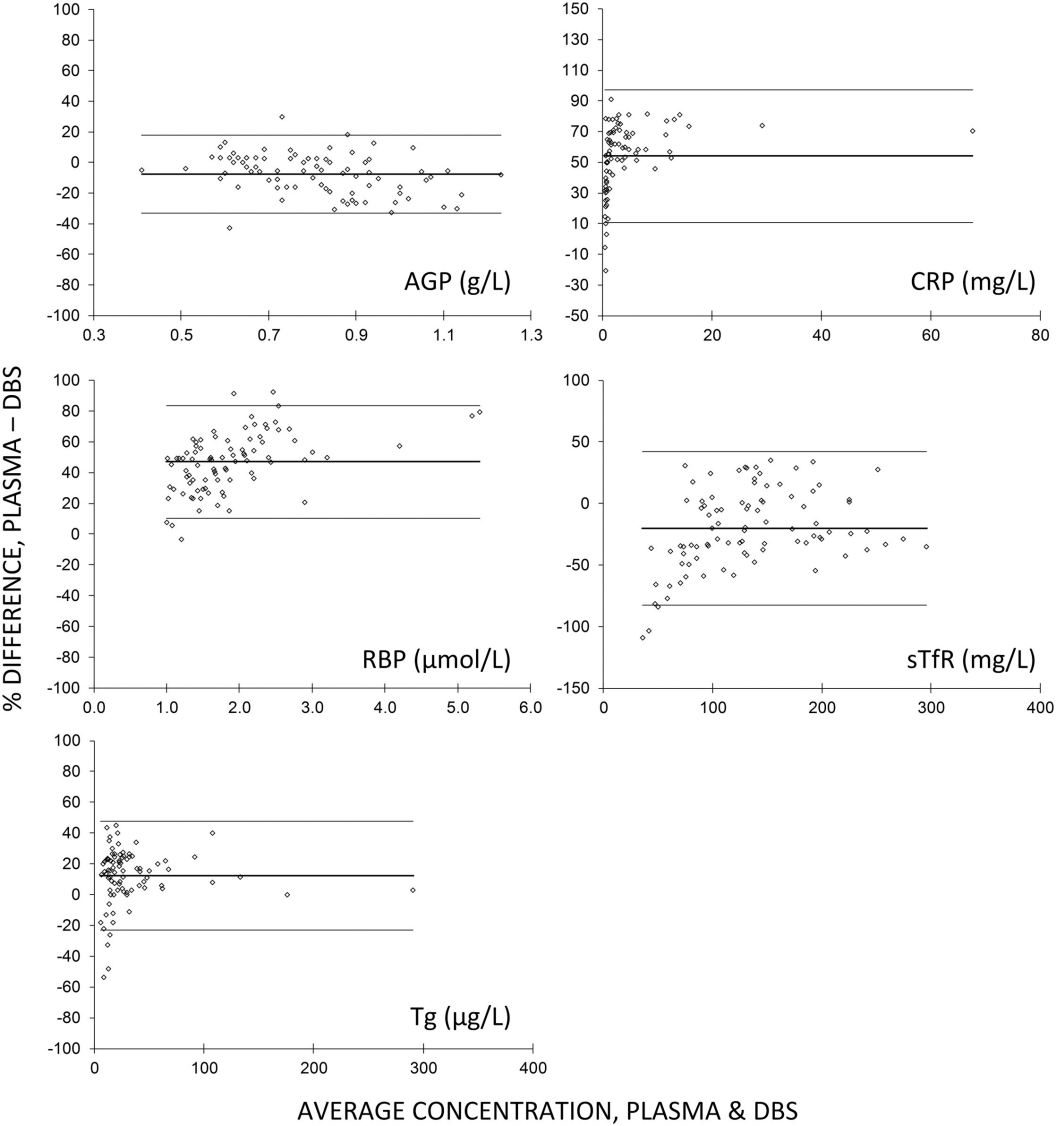
Bland Altman plots, DBS eluates versus paired plasma results derived the human micronutrient Q-plex assay. Bland-Altman plots showing percent difference between the DBS eluates and paired plasma sample results on the y-axes plotted against average concentration on the x-axes. Heavy horizontal line and light horizontal lines indicate mean ± 2standard deviations of percent difference. AGP, α-1-acid glycoprotein; CRP, C-reactive protein; RBP4, retinol binding protein 4; sTfR, soluble transferrin receptor; Tg, thyroglobulin; DBS, dried blood spot.

Spearman correlations for results from paired DBS and plasma samples, and DBS recovery of plasma values (DBS/plasma, expressed as a percent; Table 1), scatter plots with linear regression lines (Fig 1) and Bland-Altman plots (Fig 2) show that DBS and serum results are highly correlated, but DBS values tend to be lower than plasma values. The Bland-Altman plots also reveal that, particularly for CRP and Tg, most of the variability in recovery is at very low concentration ranges where physiologically small differences in value are exaggerated when plotted as percent difference.

**Table 1:** Spearman correlation of test data and mean recovery for analytes measured from paired samples of EDTA plasma and DBS. Abbreviations: AGP, α-1-acid glycoprotein; CRP, C-reactive protein; DBS, dried blood spots; N, number; RBP4, retinol-binding protein 4; SD, standard deviation; Rho, rank-order correlation; sTfR, soluble transferrin receptor; Tg, thyroglobulin.

	AGP	CRP	RBP4	sTfR	Tg
N (valid DBS and plasma pairs)	90	79	90	90	86
Correlation (Spearman’s Rho)					
Correlation coefficient	0.807	0.982	0.838	0.851	0.959
Sig(2-tailed)	<0.001	<0.001	<0.001	<0.001	<0.001
Recovery					
Mean, DBS/plasma (%)	108.8%	58.8%	62.9%	130.7%	90.1%
SD, DBS/plasma (%)	14.4%	15.6%	12.6%	48.5%	18.7%

Differences in absolute value by sample type were modest for AGP, Tg, CRP, and RBP4 and within recovery ranges common for DBS assays, and a linear regression adjustment can be applied to correct DBS results to a serum-equivalent value [19,25,26]. However, for sTfR, DBS yielded higher values than plasma, which was unexpected. Further analysis confirmed sTfR assay interference resulting from collecting blood in tubes containing EDTA; these results therefore cannot be used to determine a serum equivalent for sTfR values from DBS collected by fingerstick.

None of the blood donors in this panel had recent exposure to malaria, and therefore, there were no positive HRP2 results in the set of paired DBS and plasma specimens. Instead, HRP2 recovery from DBS was tested using washed red blood cells spiked with calibrators for all analytes at six concentrations and spotted on filter paper. Five of the six tested concentrations yielded results within the assay limits of quantification. Average recovery (observed value/expected value) for contrived DBS specimens at 8.6, 25.6, 77.2, 231.7, and 695 pg/mL of HRP2 tested on three plates was 99% +/- 28%. DBS prepared from contrived whole blood samples containing zero, 0.001%, 0.5%, and 5.0% parasitemia of *P*. *falciparum* yielded the expected dose response: the negative sample result was below the lower limit of quantification, the low dose was within the quantifiable range, and both medium and high doses had results greater than the upper limit of quantification.

## Discussion and conclusions

As part of our goal to increase the opportunity for population surveillance of micronutrient deficiencies in low- and middle-income countries we have initially assessed the capability of the Quansys 7-plex Human Micronutrient immunoarray to use DBS as an alternative specimen type to serum or plasma due to its inherent simplicity for sample collection and the redundancy of cold chain for shipping once prepared. We describe a protocol modification for use with DBS and assess its effective in comparison to paired wet plasma samples [23]. Although this protocol requires that samples are run at a final dilution of 1:20, rather than the 1:10 suggested in the kit insert, the vast majority of results were within the assay limits of quantification suggesting that the use of DBS is feasible. The primary advantage of using this multiplex panel is that this protocol requires only two punches, each 6.1 mm in diameter, for measures of six biomarkers related to MND, inflammation and malaria. Measuring each analyte individually by conventional enzyme-linked immunosorbent assay would consume a DBS sample quantity that can be difficult to obtain by fingerstick or heelstick. Simultaneous measurement also greatly reduces the assay costs, and the labor involved in DBS punching and elution.

Spearman correlations between DBS and wet plasma were high, and differences in recovered absolute values by sample type were consistent with previous DBS assays [12,15,19–22], and amenable to adjustment by linear regression, for AGP, CRP, RBP4 and Tg, and our contrived HRP2 DBS also demonstrated good agreement between sample types. However, we noted that the K_2_-EDTA anticoagulant used in preparing the paired plasma and DBS interfered with the sTfR assay component of the 7-plex array. While our initial 5-plex immunoarray indicated that all sample types (plasma derived from EDTA or heparinized samples and also serum), gave highly similar results [24], the newer 7-plex format is challenged for accurately measuring sTfR from EDTA plasma specimens [23]. The sTfR antibodies in the original assay were replaced due to supply challenges. While we were able to demonstrate that RBP4, Tg, CRP, AGP and HRP2 were all correctly measured in the 7-plex format when using EDTA plasma, further testing using a DBS panel prepared from blood collected without anticoagulant (the intended specimen type), or with heparinized whole blood, is required to derive an sTfR DBS to serum-equivalent conversion factor. A previous study has shown that heparinized plasma does not grossly affect the sTfR assay [23].

Other limitations of this test include the use of an adult US donor panel of blood specimens, which precluded evaluation of natural HRP2-positive specimens and included a limited concentration range for the MND and other biomarkers. Further evaluation is required, first to test paired serum and DBS prepared from blood samples collected without anticoagulant, and secondly, to test the array using a larger DBS specimen set collected by finger stick in a population at higher risk for MND and malaria exposure to confirm that DBS yield quantifiable results across the full physiological ranges using this multiplex array. A third component would be to access cohorts of populations at greatest risk of MND, in particular infants and young children and women of child bearing age. To enable a more complete understanding of this sampling method, comparative analyses of these samples must also include wet plasma or serum prepared from paired venous blood draws. We are currently investigating further into accessing more suitable sample sets by which to assess these. In this work we held a small prospective study with recently collected DBS samples and did not test any archived samples. We postulate that it may be feasible to perform retrospective analysis with archived DBS samples provided they were correctly prepared and stored at ultra-low temperature (-70 °C). However, while there is evidence of stability with CRP, RBP4, sTfR and Tg for up to four weeks at ambient temperatures [12,19–21,27,28], some decay (CRP, RBP4 and sTfR) was observed with longer storage at 4 °C or -20 °C [19,21,27], and with short-term exposure to extreme heat 37 °C [19]. An alternative approach is to utilize ferritin from fingerstick collection is to fractionate of the whole blood, and then apply a volume of serum onto paper to serve as a dried serum spot from which ferritin can later be assayed [29]. This can be performed via centrifugation or by devices that offer passive plasma or serum separation are less labor intensive and typically rely on capillary action for separation of the serum from the cellular components of blood. While passive separation would be of great application to surveillance, a recent assessment of nine commercial blood collection products for serum production found only one tool with acceptable criteria for use [30].

This preliminary work has however, provided a protocol for eluting samples from DBS and demonstrates that it is feasible to use DBS with the Quansys 7-plex Human Micronutrient immunoarray with samples eluted from these. The use of DBS as a sample type will enable simpler and more cost effective specimen collection to support MND surveillance in vulnerable populations in austere settings where venous blood collection is typically not a feasible option.
